# *Brassica napus* BnaC9.DEWAX1 Negatively Regulates Wax Biosynthesis via Transcriptional Suppression of *BnCER1-2*

**DOI:** 10.3390/ijms24054287

**Published:** 2023-02-21

**Authors:** Saiyu Wang, Chengcheng Bai, Na Luo, Youwei Jiang, Yulu Wang, Yu Liu, Chunjie Chen, Yuxin Wang, Qiaoqiao Gan, Shurong Jin, Yu Ni

**Affiliations:** 1 College of Agronomy, Qingdao Agriculture University, Qingdao 266109, China; 2 College of Agronomy and Biotechnology, Southwest University, Chongqing 400716, China; 3 Qingdao Key Laboratory of Specialty Plant Germplasm Innovation and Utilization in Saline Soils of Coastal Beach, Qingdao Agriculture University, Qingdao 266109, China

**Keywords:** *Brassica napus*, cuticular wax biosynthesis, *BnCER1-2*, BnaC9.DEWAX1, transcription repressor

## Abstract

Very-long-chain alkane plays an important role as an aliphatic barrier. We previously reported that *BnCER1-2* was responsible for alkane biosynthesis in *Brassica napus* and improved plant tolerance to drought. However, how the expression of *BnCER1-2* is regulated is still unknown. Through yeast one-hybrid screening, we identified a transcriptional regulator of *BnCER1-2*, BnaC9.DEWAX1, which encodes AP2\ERF transcription factor. BnaC9.DEWAX1 targets the nucleus and displays transcriptional repression activity. Electrophoretic mobility shift and transient transcriptional assays suggested that BnaC9.DEWAX1 repressed the transcription of *BnCER1-2* by directly interacting with its promoter. *BnaC9.DEWAX1* was expressed predominantly in leaves and siliques, which was similar to the expression pattern of *BnCER1-2*. Hormone and major abiotic stresses such as drought and high salinity affected the expression of *BnaC9.DEWAX1*. Ectopic expression of *BnaC9.DEWAX1* in Arabidopsis plants down-regulated *CER1* transcription levels and resulted in a reduction in alkanes and total wax loads in leaves and stems when compared with the wild type, whereas the wax depositions in the *dewax* mutant returned to the wild type level after complementation of *BnaC9.DEWAX1* in the mutant. Moreover, both altered cuticular wax composition and structure contribute to increased epidermal permeability in *BnaC9.DEWAX1* overexpression lines. Collectively, these results support the notion that BnaC9.DEWAX1 negatively regulates wax biosynthesis by binding directly to the *BnCER1-2* promoter, which provides insights into the regulatory mechanism of wax biosynthesis in *B. napus*.

## 1. Introduction

Drought is an environmental stress that limits the distribution of plants, affects plant growth and development, reduces crop productivity, and, therefore, leads to severe agroeconomic losses [1,2]. To withstand drought stress, plants have evolved many strategies to prevent water loss, to balance optimal water supply to all vital organs, and to maintain the cellular water content [2]. Land plants developed a hydrophobic cuticular wax layer that resists nonstomatal water loss, as well as various biotic and abiotic stresses [3,4,5]. Recent studies have focused on modifying cuticular waxes to improve plant tolerance to drought. For example, ectopic expression of wax-associated genes and transcription factors in transgenic plants can increase wax deposition and confer increased tolerance to water deficiency in some species [6,7,8,9]. Cuticular waxes are composed of very-long-chain fatty acids (VLCFAs) and their derivatives, such as aldehydes, alkanes, primary alcohols, secondary alcohols, ketones, and wax esters [10]. VLCFAs are elongated from C_16_ and C_18_ fatty acids by the fatty-acid elongase (FAE) complex in the endoplasmic reticulum (ER), and then converted to aldehydes, alkanes, secondary alcohols, and ketones via the alkane-forming pathway and primary alcohols and wax esters via the alcohol-forming pathway [10,11,12,13].

As the major wax compounds in Arabidopsis and many other species, alkanes contribute to cuticle characteristics associated with drought tolerance of the plant [14,15,16], and, therefore, are potential targets for crop improvement. It has been previously reported that CER1 interacts with both CER3 and CYTB5 to catalyze alkane formation, of which CER3 functions as a VLCFA-reductase-producing fatty aldehyde, whereas CER1 functions as an aldehyde-decarbonylase-producing *n*-alkane from aldehydes [14,17]. Furthermore, CER1-LIKE1 has been reported to be involved in the alkane-forming pathway with different acyl chain-length specificities from CER1 [18].

Some transcription factors involved in alkane formation have been reported in recent studies. The AP2/ERF-type transcription factor WIN1/SHN1 was first reported as a transcriptional activator that regulates cuticular wax biosynthesis and, therefore, improves plant drought tolerance; *CER1*, *CER2*, and *KCS1* genes are regulated by WIN1/SHN1 [3]. DEWAX and DEWAX2, also members of AP2/ERF subfamily, have been found to negatively regulate cuticular wax biosynthesis in Arabidopsis by directly binding to the promoters of the cuticular wax biosynthetic gene *LACS2* and *CER1* [19,20]. Two Arabidopsis MYB-SHAQKYF transcription repressors regulate leaf wax biosynthesis via transcriptional suppression on *DEWAX* [21]. Furthermore, it has been found that SPL9 activates *CER1* expression by directly binding to GTAC motifs in the *CER1* promoter; meanwhile, SPL9 antagonistically acts with DEWAX to control *CER1* expression and mediates light–dark, on–off switch-controlling wax synthesis [21]. An AP2/DREB transcription factor RAP2.4 was found to activate cuticular wax biosynthesis by increasing the expression of *KCS2* and *CER1* in Arabidopsis leaves under drought [22]. MYB transcription factors are also involved in the regulation of the alkane-formation pathway and plant tolerance to abiotic and biotic stresses. For example, MYB96 promotes wax biosynthesis in Arabidopsis by regulating the *KCS1*, *KCS2*, *KCS6*, *KCR1*, and *CER3* genes under drought stress [23], whereas MIEL1 E3 ubiquitin ligase is involved in wax biosynthesis by controlling the protein stability of MYB96 and MYB30 [24]. 

Apart from transcriptional regulation, the alkane-forming pathway is also regulated at the post-transcriptional level and epigenetic level. For example, trans-acting small interfering RNAs (tasiRNAs) directly control *CER3* expression at the post-transcriptional level and regulate stem wax deposition in Arabidopsis [25,26]. SAGL1 mediates proteasome-dependent degradation of CER3, thereby negatively regulating cuticular wax biosynthesis [27]. Histone H2B monoubiquitination has been reported to be involved in wax biosynthesis by targeting *LACS2* and *CER1* [28]. The Arabidopsis histone methyl transferases SDG8 and SDG25 contribute to wax accumulation through histone lysine methylation and/or H2B ubiquitination by targeting CER3 [29]. GCN5-mediated histone acetylation of CER3 also contributes to Arabidopsis cuticular wax biosynthesis [30].

As one of the most important oil crops worldwide, *Brassica napus* provides edible plant oil for humans and feed to animals; it also maintains soil fertility in crop rotations. Compared to other crops, *B. napus* is particularly susceptible to drought stress during the seedling and flowering stages and needs more water for its growth and development [31,32]. Therefore, improving the drought tolerance of *B. napus* has practical significance for increasing cultivation and stabilizing oilseed supply. A previous study reported that overexpression of *BnCER1-2* in *B. napus* promotes the production of alkanes and total wax and increases plant tolerance to drought [33]. However, how the expression of *BnCER1-2* in *B. napus* is regulated still remains unknown. With the goal of exploring the transcriptional regulation underlying the alkane-forming pathway in *B. napus*, we cloned the *BnCER1-2* promoter fragment containing three GCC-like motifs as bait to perform a yeast-one-hybrid assay. The *BnaC9.DEWAX1* gene, an Arabidopsis *DEWAX* ortholog in *B. napus,* was thereby identified. We revealed that BnaC9.DEWAX1 could repress the expression of the *BnCER1-2* gene via direct binding to its promoter. Furthermore, overexpression of *BnaC9.DEWAX1* in Arabidopsis inhibited alkane biosynthesis and total wax loads by down-regulating *CER1* expression. Increased cuticle permeability in *BnaC9.DEWAX1* transgenic Arabidopsis was observed and was attributed mainly to altered wax composition and crystallization in transgenic plants.

## 2. Results

### 2.1. Transcription Factor BnaC9.DEWAX1 Binds to and Represses the BnCER1-2 Promoter

*BnCER1-2* is responsible for very-long-chain alkane biosynthesis of wax in *B. napus* [33]. To better understand the regulatory mechanisms underlying *BnCER1-2* gene expression, we used a 195-bp *BnCER1-2* promoter fragment containing three GCC-like motifs as bait in a yeast-one-hybrid assay. The result showed that BnaC09g05370D could interact with the *BnCER1-2* promoter (Figure S1). Sequence analysis showed that BnaC09g05370D contained a typical AP2 DNA-binding domain and an acidic region in its N-terminus, and a predicted nuclear localization signal (NLS) in its C-terminus (Figure S2A). Phylogenetic analysis suggested that *BnaC09g05370D* was orthologous to Arabidopsis *DEWAX* (Figure S2B); therefore, it was designated as *BnaC9.DEWAX1*.

**Figure 1:**
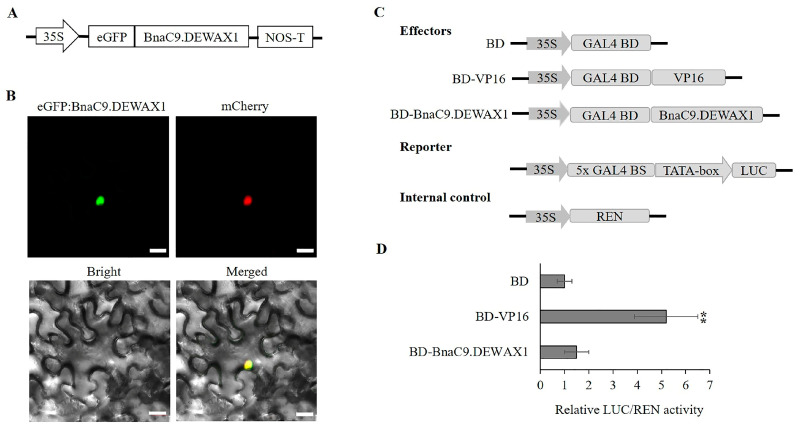
Subcellular localization and transcriptional activity analysis of BnaC9.DEWAX1 in tobacco leaf and Arabidopsis protoplasts. (**A**) Schematic diagram of eGFP:BnaC9.DEWAX1 construct used for Subcellular localization. (**B**) Confocal images of tobacco leaf transient expressing eGFP:BnaC9.DEWAX1. The nuclear marker mCherry-D53 was used as a positive control. Bar = 20 μm. (**C**) Schematic diagram of effector and reporter constructs used for the BnaC9.DEWAX1 transcriptional activity assay. BD—GAL4 DNA-binding domain. VP16—herpes virus protein VP16 activation domain. (**D**) Transcriptional activity assay of BnaC9.DEWAX1 in Arabidopsis leaf protoplasts. Arabidopsis leaf protoplasts were transformed with the reporter and effector constructs. Relative ratios of LUC/REN were used to evaluate the transcriptional activity of BnaC9.DEWAX1. Error bars indicate ± SD from three independent experiments. Data were statistically analyzed using Student’s *t* test (** *p* < 0.01).

Transient transfection of tobacco leaf with the cauliflower mosaic virus (CaMV) *35S* promoter-driven eGFP:BnaC9.DEWAX1 construct showed that BnaC9.DEWAX1 was localized in the nucleus (Figure 1A,B), which was consistent with the predicted NLS in BnaC9.DEWAX1. To determine whether BnaC9.DEWAX1 harbors transcriptional activity, an effector construct which harbors BnaC9.DEWAX1 and the Gal4 DNA-binding domain (BD) and a reporter construct which contains a Gal4-binding site (BS) and a CaMV *35S* minimal promoter-driven luciferase (LUC) were used in a transactivation assay with Arabidopsis protoplasts. Two other effectors, a BD vector and a VP16 vector which harbors a transcriptional activation domain, were used as control in transactivation assay (Figure 1C). As shown in Figure 1D, BnaC9.DEWAX1 was able to suppress 50% transcriptional activation by VP16. These results suggested that BnaC9.DEWAX1 could function as a transcriptional repressor.

**Figure 2:**
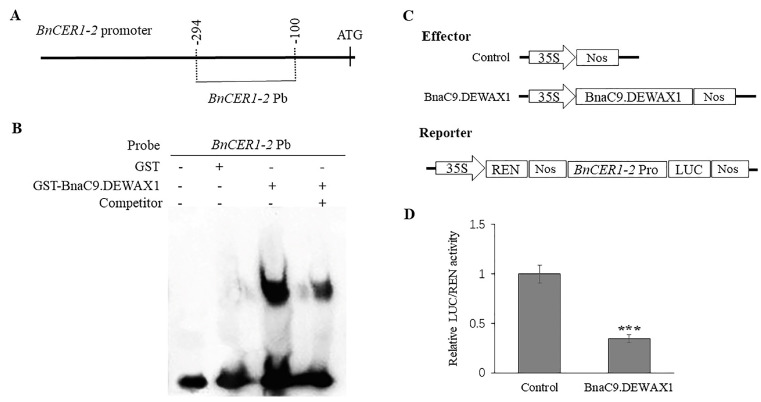
BnaC9.DEWAX1 binds to *BnCER1-2* promoter and acts as a transcriptional repressor. (**A**) Diagram of the *BnCER1-2* promoter fragment used for EMSA. The position of the promoter fragment relative to the start codon is shown above the line. (**B**) EMSA of BnaC9.DEWAX1 binding to the promoter sequence of *BnCER1-2* gene. Labeled DNA fragment (50 µM) incubated with 6 μg of recombinant GST-BnaC9.DEWAX1 protein was assayed. GST was used as a control protein. For competition analysis, excess unlabeled DNA fragments relative to the labeled probes were included in the reactions. (**C**) Diagram of reporter and effector constructs used for transactivation analysis. (**D**) BnaC9.DEWAX1 transcriptionally repressed *BnCER1-2* promoter-driven expression of LUC reporter gene. The reporter and effector construct were co-transfected into Arabidopsis leaf protoplasts. LUC activity was measured and normalized based on the level of REN activity. Error bars represent SD of three biological replicates. Data were statistically analyzed using Student’s *t* test (*** *p* < 0.001).

To further confirm that BnaC9.DEWAX1 binds to the *BnCER1-2* promoter, we expressed the BnaC9.DEWAX1 protein as a GST fusion in *E. coli*, and the purified recombinant proteins were then used for an electrophoretic mobility shift assay (EMSA). Meanwhile, a 195-bp *BnCER1-2* promoter fragment used in the yeast-one-hybrid assay was labeled for EMSA (Figure 2A). As shown in Figure 2B, the recombinant BnaC9.DEWAX1 protein was able to bind to the *BnCER1-2* promoter fragment and caused a retarded band. In contrast, no retarded band was detected when the *BnCER1-2* promoter fragment was incubated with GST protein alone. The addition of an unlabeled *BnCER1-2* promoter fragment weakened the retarded band due to competition with the binding (Figure 2B). These results suggested that the BnaC9.DEWAX1 protein could bind to the *BnCER1-2* promoter fragment. 

In subsequent co-transfection of Arabidopsis leaf protoplasts with the *CaMV 35S* promoter-driven *BnaC9.DEWAX1* expression construct and the *BnCER1-2* promoter-driven LUC reporter gene, the expression of *BnaC9.DEWAX1* resulted in a substantial reduction in the expression of the reporter gene LUC, indicating that BnaC9.DEWAX1 was able to transcriptionally repress the expression of *BnCER1-2* promoter (Figure 2C,D).

### 2.2. BnaC9.DEWAX1 Is Predominately Expressed in the Leaf and Exhibits Altered Levels of Expression in Response to Abiotic Stress

**Figure 3:**
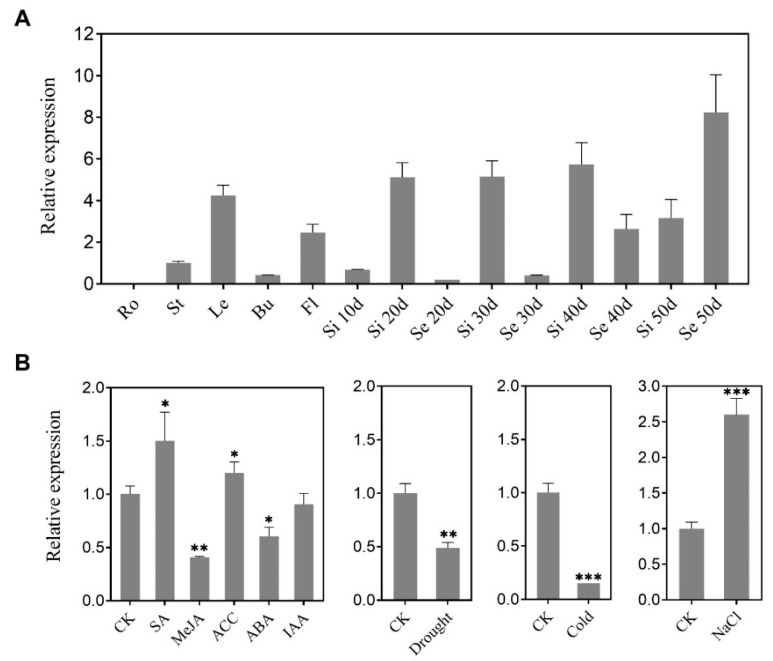
Expression pattern of *BnaC9.DEWAX1* gene. (**A**) qRT-PCR analysis of *BnaC9.DEWAX1* expression in various *Brassica napus* organs. Ro—root; St—stem; Le—leaf; Bu—bud; Fl—flower, Si 10–50d, siliques 10–50 days after flowering; Se 20–50d, seeds 20–50 days after flowering. (**B**) qRT-PCR analysis of *BnaC9.DEWAX1* expression under abiotic stresses. Leaves were harvested from eight-week-old plants at 6 h after incubation in a solution containing 100 μM SA, 10 μM MeJA, 10 μM ACC, 10 μM ABA, 1 μM IAA, or 150 mM NaCl. For drought stress, plants were air-dried for 6 h. For cold stress, plants were placed in a cold room (4 °C) for 6 h. Leaves from plants without treatments were used as control. Total RNA was extracted from *B. napus* leaves and other organs for qRT-PCR. Abundance of the *BnActin 7* transcripts in each sample was used to normalize differences in the total RNA amount. Error bars indicate ± SD from three biological replicates. Data were statistically analyzed using Student’s *t* test (* *p* < 0.05; ** *p* < 0.01; *** *p* < 0.001).

To investigate the expression of the *BnaC9.DEWAX1* gene in *B. napus* and its response to various abiotic stresses, total RNA was isolated from various *B. napus* organs and leaves treated by abiotic stresses and subjected to quantitative RT-PCR. The *BnaC9.DEWAX1* gene was strongly expressed in leaves, siliques, and late developmental seeds, whereas it was least expressed in roots and early-stage seeds (Figure 3A). The expression level of *BnaC9.DEWAX1* was up-regulated in response to SA, ACC, and NaCl stress, whereas it was down-regulated by MeJA, ABA, drought, and cold stresses (Figure 3B).

### 2.3. Cuticular Wax Deposition Is Negatively Regulated by BnaC9.DEWAX1

**Figure 4:**
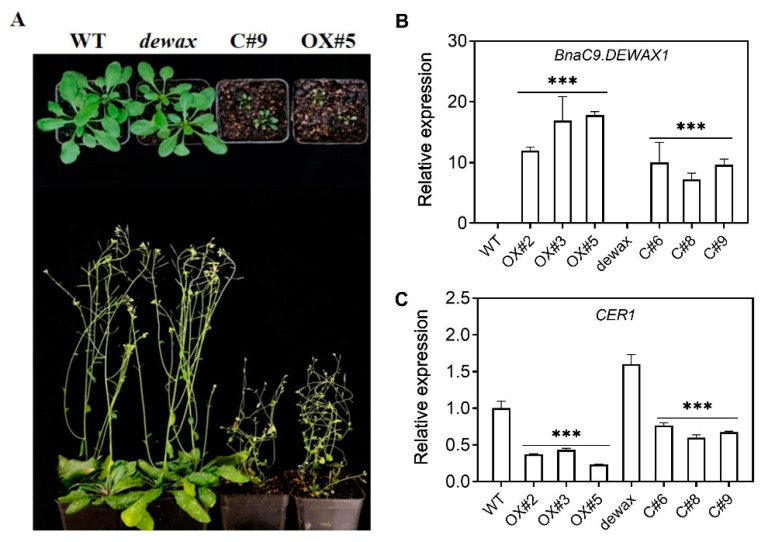
The transcription level of *BnaC9.DEWAX1* and *CER1* in Arabidopsis and phenotypic characterization of *BnaC9.DEWAX1* transgenic plants. (**A**) Phenotype of Arabidopsis wild type and *BnaC9.DEWAX1* overexpression and complementation lines. (**B**) Increased transcription level of *BnaC9.DEWAX1* in overexpressing and complementation lines relative to wild type. (**C**) Down-regulation of *CER1* involved in cuticular wax biosynthesis by BnaC9.DEWAX1. Total RNA was extracted from the leaves of four-week-old Arabidopsis. Error bars indicate ± SD from three biological replicates. Data were statistically analyzed using Student’s *t*-test (*** *p* < 0.001).

The transcriptional repression of BnaC9.DEWAX1 on the expression of *BnCER1-2* prompted us to investigate whether BnaC9.DEWAX1 regulated VLC-alkane biosynthesis and wax load. We generated transgenic Arabidopsis lines that overexpress *BnaC9. DEWAX1* under the control of the CaMV *35S* promoter in the wild type and the *dewax* mutant, respectively. The positive transgenic lines were identified by amplification of an 35S:BnaC9.DEWAX1 fragment and hygromycin antibiotic resistance. Compared with the WT and mutant, the growth and development of transgenic plants were delayed to varying degrees (Figure 4A). qRT-PCR analysis showed that the expression of *BnaC9.DEWAX1* increased significantly in the overexpression lines OX#2, OX#3, and OX#5 (Figure 4B), whereas the expression of *CER1* was decreased by approximately 57–77% in overexpressing lines relative to the WT (Figure 4C). Relative to the *dewax*, the expression of *BnaC9.DEWAX1* in the complementation lines C#4, C#6, and C#9 increased significantly, whereas the expression of *CER1* decreased significantly in complementation lines (Figure 4B,C). These results suggested that BnaC9.DEWAX1 negatively regulates the expression of *CER1* involved in Arabidopsis cuticular wax biosynthesis.

**Figure 5:**
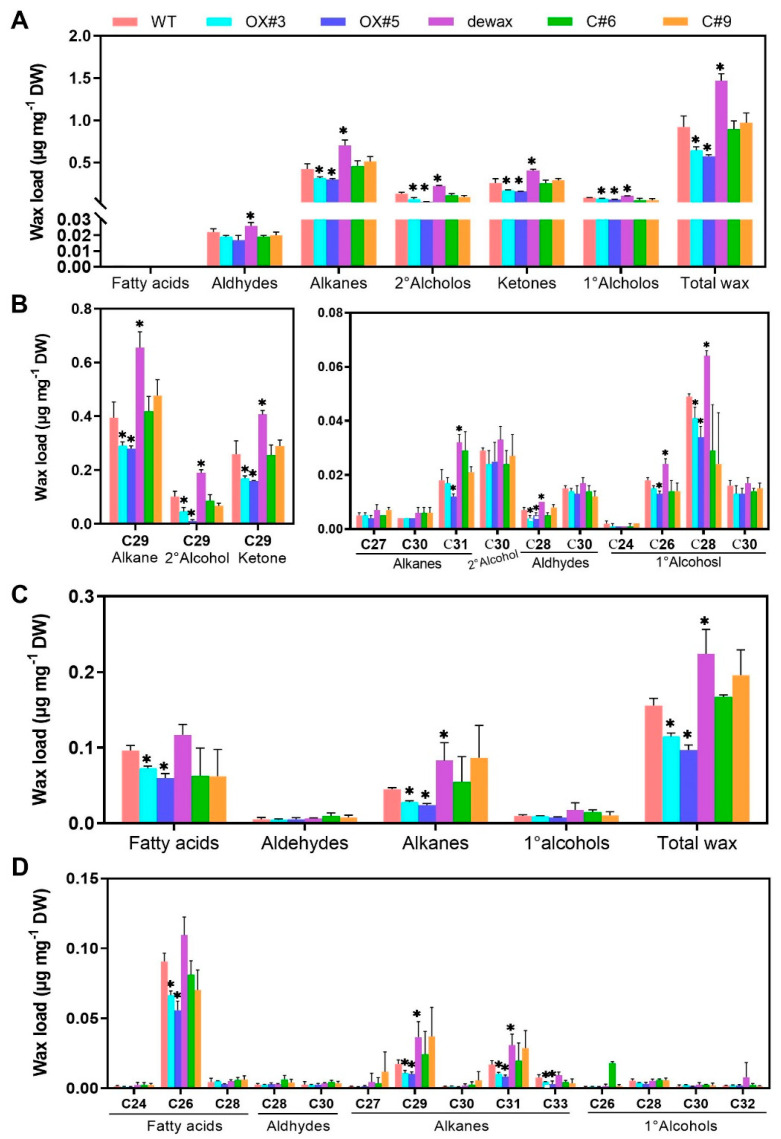
Cuticular wax analysis in Arabidopsis wild type and *BnaC9.DEWAX1* overexpression and complementation lines. (**A**) Total wax coverage and amount of each wax class in stem. (**B**) Amounts of individual components in each wax class in stem. (**C**) Total wax coverage and amount of each wax class in leaf. (**D**) Amounts of individual components in each wax class in leaf. Cuticular waxes were extracted from six-week-old plants with chloroform and analyzed using gas chromatography. Error bars indicate ± SD from three biological replicates. Data were statistically analyzed using Student’s *t*-test (* *p* < 0.05).

Overexpression of *BnaC9.DEWAX1* in *A. thaliana* WT significantly reduced the contents of alkanes, secondary alcohols, ketones, primary alcohols, and total wax on stems (Figure 5A), and the contents of fatty acids, alkanes, and total wax on leaves (Figure 5C). For wax components, *BnaC9.DEWAX1* gene overexpression mainly decreased the contents of C_29_ alkanes, C_29_ secondary alcohol, and C_29_ ketones, the three predominant compounds in total wax, as well as C_28_ aldehyde and C_28_ primary alcohols on the stem (Figure 5B). For leaf, *BnaC9.DEWAX1* gene overexpression mainly decreased the contents of C_26_ fatty acid and C_29_, C_31_, and C_33_ alkanes (Figure 5D). As described previously [19], the *dewax* mutant plants showed higher contents of wax compositions and total wax on both stem and leaves compared with the WT. When the *BnaC9.DEWAX1* gene driven by the CaMV *35S* promoter was expressed in Arabidopsis *dewax* plants, no significant differences in wax depositions on stems and leaves were observed between the complementation lines and the WT (Figure 5).

### 2.4. BnaC9.DEWAX1 Negatively Regulates Cuticle Permeability in Arabidopsis

**Figure 6:**
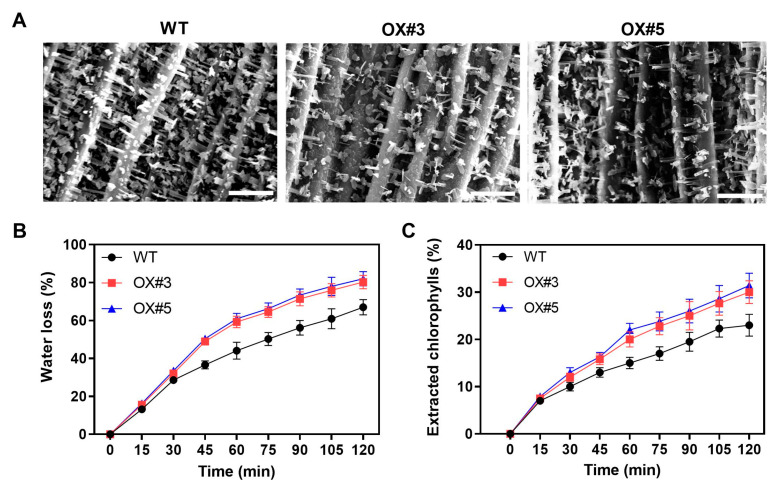
Epicuticular wax crystal structure and cuticle permeability in WT and *BnaC9.DEWAX1* overexpression lines. (**A**) Scanning electron microscope images of epicuticular waxes on the top of inflorescence stem surface. Scale bars = 10 µm. (**B**) Water-loss rates of isolated six-week-old rosettes. (**C**) Chlorophyll extraction rates of isolated six-week-old rosettes. Error bars indicate ± SD of five replicates.

To examine whether altered amount of cuticular wax of *BnaC9.DEWAX1* overexpression lines affected plant wax structure and cuticle permeability, we compared the epicuticular wax morphology between WT and overexpression lines. As Figure 6A shown, the overexpression lines showed reduced crystals on stems when compared with the WT. Next, we evaluated leaf cuticle permeability by water loss measurement and chlorophyll extraction. Compared with the WT, the leaves from the overexpression lines showed increased water loss rate and extracted chlorophylls rate (Figure 6B,C), suggesting that overexpression of *BnaC9.DEWAX1* led to increased cuticle permeability.

## 3. Discussion

Cuticular waxes covering the plant’s outermost layer serve as the first protection barrier against environmental stresses, and its biosynthesis is tightly regulated by development and environmental factors [4]. However, the genetic control of wax biosynthesis in *B. napus* is less understood. *B. napus* is one of the most important oil crops worldwide. During the seedling and flowering stage, *B. napus* is sensitive to water shortage. We previously reported that overexpression of *BnCER1-2* in *B. napus* promotes the production of alkanes and total wax and increases plant tolerance to drought [33]. *BnCER1-2* is homologous to Arabidopsis CER1 and functions as an aldehyde decarbonylase producing *n*-alkanes from aldehydes [14]. Nonetheless, how the expression of *BnCER1-2* in *B. napus* is regulated remains unknown. Transcriptional regulation is one of the most important strategies for regulating plant stress responses [34]. In this study, we identified an ERF-type transcription factor BnaC9.DEWAX1, one new component in *B. napus* wax biosynthesis. Sequence and phylogenetic analysis suggested that BnaC9.DEWAX1 is a member of the ERF subfamily B-3 of ERF/AP2 transcription factor family and is homologous to Arabidopsis DEWAX. Arabidopsis DEWAX is reported to bind to the classic GCC motifs or variants in the promoters of wax genes such as *CER1*, *FAR6*, *LACS2*, *ACLA2*, and *ECR* [19]. As shown in Figure 2 and Figure S1, BnaC9.DEWAX1 suppressed *BnCER1-2* expression by directly binding on its promoter regions that harbor three GCC-like motifs. Transcription activity analysis suggested that BnaC9.DEWAX1 was a transcriptional repressor, though no EAR repression motif was found in BnaC9.DEWAX1. These results suggest that BnaC9.DEWAX1-mediated repression of *BnCER1-2* transcription may contribute to the wax deposition in *B. napus*. *BnaC9.DEWAX1* was predominantly expressed in leaves, siliques, and late developmental seeds but was not detected in roots (Figure 3A), which was similar to the expression pattern of *BnCER1-2* being in mainly leaves and siliques [33], suggesting that BnaC9.DEWAX1 may be involved in the biosynthesis of leaf and silique wax.

To prove the function of BnaC9.DEWAX1 in wax biosynthesis, transgenic Arabidopsis lines overexpressing *BnaC9.DEWAX1* and complementation lines were generated. The *CER1* expression levels were correlated with the altered wax loads in *BnaC9.DEWAX1* overexpression and complementation plants (Figure 4). *BnaC9.DEWAX1* gene overexpression mainly reduced the contents of C_29_ alkanes, C_29_ secondary alcohol, and C_29_ ketones, the three predominant compounds produced from the alkane-forming pathway, thereby reducing the total wax loads (Figure 5A,B). This mechanism could be due to down-regulated expression of *CER1*, which is responsible for alkane biosynthesis (Figure 4C). As reported previously [19], the Arabidopsis *dewax* mutant showed an increase in total leaf and stem wax loads. In this study, the excess wax phenotype of dewax was restored to wild type levels by overexpressing *BnaC9.DEWAX1* in *dewax* mutant. Both promoters of *BnCER1-2* and *CER1* contain a GCC-like motif (Table S1). These results suggested that BnaC9.DEWAX1 negatively regulated alkane/wax biosynthesis, and this effect was achieved by inhibiting the expression of *CER1*/*BnCER1-2*.

The overexpression of the *BnaC9.DEWAX1* gene in Arabidopsis also reduced cuticle permeability (Figure 6B,C). Quantitative analysis of cuticular wax revealed that the amounts of long-chain alkanes and total waxes in both the leaf and stem of overexpressing lines were significantly lower than that in wild type plants (Figure 5). Currently, *n*-alkanes are one of the few wax components with an established specific contribution to cuticle properties. The accumulation of the *n*-alkanes and the reduction in cuticle permeability led to a better plant resistance to water stress [14,16,35]. Excepting the reduction in alkanes and total wax loads, overexpression of *BnaC9.DEWAX1* also resulted in a reduction in the density of wax crystals on Arabidopsis stem (Figure 6A). The formation of three-dimensional epicuticular wax crystallites can significantly increase contact angles, which renders leaf surfaces essentially non-wettable [36]. Thus, both altered cuticular wax composition and structure contribute to increased epidermal permeability in *BnaC9.DEWAX1* overexpression lines. Additionally, altered expression levels of *BnaC9.DEWAX1* under abiotic stress and hormone treatments indicated the role of *BnaC9.DEWAX1* in responses of plants to major abiotic stresses such as drought and high salinity (Figure 3B). Overall, these results suggested that the alkane synthesis was regulated by BnaC9.DEWAX1-BnCER1-2/CER1 module, which controlled water loss and played an important role in plant response to water deficiency. 

In summary, we identified a transcription repressor BnaC9.DEWAX1, which negatively regulated wax biosynthesis by suppressing *BnCER1-2* expression. The results provide insight into understanding the regulatory mechanisms controlling cuticular wax accumulation in *B. napus* and also provide a promising target gene for improving the drought tolerance of rapeseed.

## 4. Materials and Methods

### 4.1. Plant Materials and Growth Conditions

*B. napus* cultivars Zhongshuang11 (ZS11) were used for gene cloning and gene expression analysis. The Arabidopsis T-DNA insertion mutant *dewax* (SALK_015182C) was obtained from the ABRC (http://www.arbidopsis.org; accessed on 31 March 2021). All plants (Arabidopsis, *B. napus*, *Nicotiana tabacum*) were grown on agar plates or in soil in a growth chamber (16 h light and 8 h darkness at 22 °C).

To examine the effects of growth hormones and stress conditions on gene expression, leaves were harvested from eight-week-old plants (ZS11) at 6 h after incubation in a solution containing 100 μM SA, 10 μM MeJA, 10 μM ACC, 10 μM ABA, 1 μM IAA, or 150 mM NaCl. For drought stress, plants were air-dried for 6 h. For cold stress, plants were placed in a cold room (4 °C) for 6 h. Leaves from plants without treatments were used as control.

### 4.2. Generation of Transgenic Plants

To generate transgenic Arabidopsis overexpressing *BnaC9.DEWAX1*, the *BnaC9.DEWAX1* coding sequence was amplified from *B. napus* using gene-specific primers (Table S2) and inserted between *Bam*HI and *Hin*dIII sites of the pC1301-DsRED vector with the CaMV *35S* promoter via homologous recombination. The recombinant vector was transformed into Arabidopsis wild type and mutant via the Agrobacterium-mediated floral-dip method [37]. Transgenic seeds were selected via fluorescence of a DsRed marker [38]. Furthermore, transgenic homozygous lines were selected via Hygromycin antibiotic resistance and verified via PCR genotyping from the T2 generation and confirmed in the T3 generation.

### 4.3. qRT-PCR Analysis

Relative expression of genes was determined using quantitative realtime PCR. Total RNA was extracted from samples using a Total RNA Extraction Kit (Promega), and then 1.5 μg total RNA was reverse transcribed to first-strand cDNA using cDNA Synthesis SuperMix (Transgen). qRT-PCR was performed using a CFX96 System (Bio-Rad) with SYBR Premix Ex Taq (TaKaRa) according to the manufacturer’s instructions. For the real-time PCR, the following program was used: 95 °C for 30 s, 45 cycles of 95 °C 10 s, 58 °C for 30 s, and 65 °C for 10 s. The expression levels of target genes were normalized to that of *EF1a* in Arabidopsis and *BnActin 7* in *B.napus*, respectively. The primer pairs used for real-time PCR are listed in Table S2. Three biological and three technical replicates were carried out for each sample.

### 4.4. Yeast One-Hybrid Assays

To perform yeast one-hybrid assays, a 195-bp *BnCER1-2* promoter fragment containing three GCC-like motifs was cloned into the pAbAi bait vector, then transformed to Y1HGold yeast competent cell to generate a bait-specific reporter strain. The *BnaC9.DEWAX1* coding sequence was cloned into the pGADT7 vector which harbors the GAL4 transcription activation domain (GAL4 AD) and then transformed to the Y1H Gold bait reporter strain. The colonies that can grow on synthetic defined (SD) medium in the absence of leucine (Leu) and containing AbA antibiotic were classified as protein–DNA interactions. Furthermore, the obtained yeast colonies were resuspended in water at equal densities, then serially spotted onto SD medium (-Leu/AbA) to check differential growth. pGADT7-53 was transformed into a p53-AbAi reporter strain as positive control, whereas it was transformed into the reporter strain containing the *BnCER1-2* promoter fragment as negative control.

### 4.5. Subcellular Localization

The *BnaC9.DEWAX1* coding sequence was inserted between *Eco*RI and *Bam*HI sites of the pBI121 vector, which harbors the CaMV *35S* promoter, eGFP, and the terminator of the nopaline synthase (Nos) gene. The generated eGFP:BnaC9.DEWAX1 plasmid was co-transfected with the nuclear marker mCherry-D53 into leaves of Nicotiana tabacum, as described previously [39]. The nuclear marker OsD53 fused with mCherry was used as positive control [40]. The GFP fluorescence was observed via a Zeiss LSM 780 confocal laser scanning microscope (Carl Zeiss, Germany) after incubation in the dark for 24 h.

### 4.6. Transcriptional Repression Assay

The activity of the BnaC9.DEWAX1 transcription factor was investigated using a transient expression system with Arabidopsis protoplasts. The *BnaC9.DEWAX1* coding sequence was fused with the GAL4 DNA-binding domain (GAL4 BD) under the control of the 35S promoter to produce the 35S:BD-BnaC9.DEWAX1 effector construct. The 35S:BD-VP16 construct, which fuses the herpes virus protein VP16 activation domain to the GAL4 BD, and the empty 35S:BD construct were separately used as positive or negative control. The reporter construct harbors a LUC gene driven by the minimal TATA box plus five GAL4-binding elements and the CaMV 35S promoter. The pRL-TK vector, which harbors the Renilla luciferase gene driven by the CaMV 35S promoter, was used as an internal control. The effector, reporter, and internal control were simultaneously transformed into the Arabidopsis leaf protoplast cells, then kept in the dark for 16 h. The activities of LUC and REN were measured with a Dual-Luciferase Reporter Assay System and luminescence reader (GROMAX-20/20, Promega). Relative ratios of LUC/REN were used to evaluate the transcriptional activity of BnaC9.DEWAX1. The primers used are listed in Supplementary Table S1.

### 4.7. Transient Dual-LUC Assay

To analyze the transcriptional repression activity of BnaC9.DEWAX1 on the promoter of the *BnaCER1-2* gene, the *BnaC9.DEWAX1* coding sequence was cloned into the pSKII plant expression vector containing the *35S* promoter and Nos terminator. The same *BnaCER1-2* promoter fragment used in the Y1H assay was cloned in the reporter vector pGreenII 0800-LUC under the control of the *35S* promoter. Then, the reporter construct was transfected into the Arabidopsis protoplast together with an effector construct or empty pSKII vector. The transformed protoplasts were incubated under dark conditions for 14–18 h. The dual-luciferase assay was performed as described by the manufacturer (Dual-Luciferase^®^ Reporter Assay, Promega, Madison, WI, USA), and the ratio of LUC/REN activity was detected using a multimode microplate reader (GloMax-20/20, Promega). Primers are listed in Supplemental Table S2.

### 4.8. Electrophoretic Mobility Shift Assays (EMSA)

The BnaC9.DEWAX1 fused with GST was expressed in *E. coli* at 18 °C for 8 h with the induction of 0.1 mM isopropyl-beta-D-thiogalactopyranoside (IPTG), and the recombinant protein was purified using Glutathione Sepharose beads according to the manufacturer’s procedure. The 195-bp *BnaCER1-2* promoter fragment which contains three GCC-like motifs that was used in the Y1H assay was labeled with biotin and then incubated with purified GST-BnaC9.DEWAX1 protein for 30 min at room temperature using the Chemiluminescent EMSA Kit (Beyotime, Shanghai, China). The reaction mixtures were separated by 6% native polyacrylamide gel electrophoresis and transferred to a nylon membrane (0.45 µm). After the membrane is crosslinked, the biotin end-labeled DNA is detected by Chemiluminescence.

### 4.9. Cuticular Wax Analysis

The extraction of Arabidopsis cuticular wax was performed as previously described with minor modifications [19]. Briefly, cuticular waxes were extracted from the stem and rosette leaves of six-week-old Arabidopsis plants by immersing them for 30 s in 5 mL chloroform solution containing 10 μg tetracosane as an internal standard. The extracts were transferred to vials; dried under nitrogen gas; derivatized by adding 20 μL of N, N-bis-trimethylsilyltrifluoroacetamide (BSTFA) and 20 μL of pyridine; and incubated for 45 min at 70 °C. Wax components were identified by GC-MS and quantified via gas chromatograph coupled to a flame ionization detector (GC–FID). The GC analysis was carried out with a 9790II gas chromatograph (Fu-Li, Hangzhou, China). The GC column was a DM-5 capillary column (30 m × 0.32 mm × 0.25 µm). Nitrogen served as the carrier gas. The GC oven was held at 80 °C for 10 min, heated at 5 °C/min to 260 °C, where the temperature remained 10 min. The temperature was then heated at 2 °C/min to 290 °C, and further heated at 5 °C/min to 320 °C, where the temperature was held for 10 min. Compounds were detected with a GCMS-QP2010 Ultra Mass Spectrometric Detector (Shimadzu Corp., Kyoto, Japan) using an HP-5 MS capillary column (30 m × 0.32 mm × 0.25 µm), and He served as the carrier gas. Compounds were identified by comparing their mass spectra with published data and authentic standards. Three biological replicates per genotype were performed. Five plants were used for each replicate. The amounts of wax were expressed per dry weight (µg mg^−1^).

### 4.10. Scanning Electron Microscope (SEM)

To view the epicuticular waxes, air-dried inflorescence stem sections from six-week-old Arabidopsis plants were mounted onto standard aluminum stubs, sputter-coated with gold particles with a Polaron SC-500 (Quorum, Lewes, UK), then viewed with a Quanta 200 microscope (FEI, Eindhoven, The Netherlands).

### 4.11. Cuticle Permeability Analysis

Cuticle permeability was measured as described previously with minor modifications [35]. The six-week-old plants were dark acclimated for 3 h to ensure stomatal closure, and then the leaves were immersed into distilled water for 1 h and weighed. Next, the leaf samples were placed into a dark chamber for continuous dehydration, and their weights were determined at 15 min intervals for 120 min to record the water loss. Then, leaf samples were dried at 70 °C for 24 h and weighed. The water loss percentage was calculated as follows: Water loss (%) = (saturated weight—fresh weight)/ (saturated weight—dry weight) × 100%. For the chlorophyll leaching assay, leaves were detached, weighed, and soaked in 80% ethanol with shaking at 40 rpm at room temperature [41]. Aliquots of 500 μL were drawn from the solution at the individual time points and quantified for extracted chlorophyll amount by measuring the absorbance at 647 and 664 nm using an ultraviolet (UV) DU7300 spectrophotometer. Total micromoles of chlorophyll = 7.93 (A_664_) + 19.53 (A_647_). Data are expressed as percentages of the total chlorophyll extracted after 24 h in 80% ethanol. Five biological replicates per genotype were performed, and three plants were used for each replicate.

### 4.12. Phylogenetic Analysis

BnaC9.DEWAX1 and the ERF subfamily proteins in Arabidopsis were aligned with ClustalX 2.1, and then the phylogenetic tree was generated using the neighbor-joining (N-J) method via MEGA 7.0 with bootstrap values of 1000 trials [42].

### 4.13. Statistical Analysis

Statistical analysis was performed using the Student’s *t*-test (* *p* < 0.05; ** *p* < 0.01; *** *p* < 0.001). The data were presented as means  ±  SD of at least three biological replicates.

## Data Availability

The data presented in this study are available in this article and its Supplementary Materials.

## Supplementary Materials

The following supporting information can be downloaded at: https://www.mdpi.com/article/10.3390/ijms24054287/s1.

## References

[1] Dietz K.J., Zorb C., Geilfus C.M. (2021). Drought and crop yield. Plant Biol..

[2] Gupta A., Rico-Medina A., Cano-Delgado A.I. (2020). The physiology of plant responses to drought. Science.

[3] Aharoni A., Dixit S., Jetter R., Thoenes E., van Arkel G., Pereira A. (2004). The SHINE clade of AP2 domain transcription factors activates wax biosynthesis, alters cuticle properties, and confers drought tolerance when overexpressed in Arabidopsis. Plant Cell.

[4] Lewandowska M., Keyl A., Feussner I. (2020). Wax biosynthesis in response to danger: Its regulation upon abiotic and biotic stress. New Phytol..

[5] Shepherd T., Griffiths D.W. (2006). The effects of stress on plant cuticular waxes. New Phytol..

[6] Al-Abdallat A.M., Al-Debei H.S., Ayad J.Y., Hasan S. (2014). Over-Expression of SlSHN1 Gene Improves Drought Tolerance by Increasing Cuticular Wax Accumulation in Tomato. Int. J. Mol. Sci..

[7] Zhang J.-Y., Broeckling C.D., Sumner L.W., Wang Z.-Y. (2007). Heterologous expression of two Medicago truncatula putative ERF transcription factor genes, WXP1 and WXP2, in Arabidopsis led to increased leaf wax accumulation and improved drought tolerance, but differential response in freezing tolerance. Plant Mol. Biol..

[8] Zhou X., Jenks M.A., Liu J., Liu A., Zhang X., Xiang J., Zou J., Peng Y., Chen X. (2014). Overexpression of Transcription Factor OsWR2 Regulates Wax and Cutin Biosynthesis in Rice and Enhances its Tolerance to Water Deficit. Plant Mol. Biol. Report..

[9] Zhu L., Guo J., Zhu J., Zhou C. (2014). Enhanced expression of EsWAX1 improves drought tolerance with increased accumulation of cuticular wax and ascorbic acid in transgenic Arabidopsis. Plant Physiol. Biochem..

[10] Samuels L., Kunst L., Jetter R. (2008). Sealing plant surfaces: Cuticular wax formation by epidermal cells. Annu. Rev. Plant Biol..

[11] Jetter R., Kunst L., Samuels A.L. (2006). Composition of Plant Cuticular Waxes. Annual Plant Reviews Volume 23: Biology of the Plant Cuticle.

[12] Kunst L., Samuels A.L. (2003). Biosynthesis and secretion of plant cuticular wax. Prog. Lipid Res..

[13] Lee S.B., Suh M.C. (2015). Advances in the understanding of cuticular waxes in *Arabidopsis thaliana* and crop species. Plant Cell Rep..

[14] Bourdenx B., Bernard A., Domergue F., Pascal S., Léger A., Roby D., Pervent M., Vile D., Haslam R.P., Napier J.A. (2011). Overexpression of Arabidopsis ECERIFERUM1 promotes wax very-long-chain alkane biosynthesis and influences plant response to biotic and abiotic stresses. Plant Physiol..

[15] Li H., Guo Y., Cui Q., Zhang Z., Yan X., Ahammed G.J., Yang X., Yang J., Wei C., Zhang X. (2020). Alkanes (C29 and C31)-Mediated Intracuticular Wax Accumulation Contributes to Melatonin- and ABA-Induced Drought Tolerance in Watermelon. J. Plant Growth Regul..

[16] Wang W., Zhang Y., Xu C., Ren J., Liu X., Black K., Gai X., Wang Q., Ren H. (2015). Cucumber ECERIFERUM1 (CsCER1), which influences the cuticle properties and drought tolerance of cucumber, plays a key role in VLC alkanes biosynthesis. Plant Mol. Biol..

[17] Bernard A., Domergue F., Pascal S., Jetter R., Renne C., Faure J.D., Haslam R.P., Napier J.A., Lessire R., Joubès J. (2012). Reconstitution of plant alkane biosynthesis in yeast demonstrates that Arabidopsis ECERIFERUM1 and ECERIFERUM3 are core components of a very-long-chain alkane synthesis complex. Plant Cell.

[18] Pascal S., Bernard A., Deslous P., Gronnier J., Fournier-Goss A., Domergue F., Rowland O., Joubès J. (2019). Arabidopsis CER1-LIKE1 Functions in a Cuticular Very-Long-Chain Alkane-Forming Complex. Plant Physiol..

[19] Go Y.S., Kim H., Kim H.J., Suh M.C. (2014). Arabidopsis Cuticular Wax Biosynthesis Is Negatively Regulated by the DEWAX Gene Encoding an AP2/ERF-Type Transcription Factor. Plant Cell.

[20] Kim H., Go Y.S., Suh M.C. (2018). DEWAX2 Transcription Factor Negatively Regulates Cuticular Wax Biosynthesis in Arabidopsis Leaves. Plant Cell Physiol..

[21] Liu Q., Huang H., Chen Y., Yue Z., Wang Z., Qu T., Xu D., Lü S., Hu H. (2022). Two Arabidopsis MYB-SHAQKYF transcription repressors regulate leaf wax biosynthesis via transcriptional suppression on DEWAX. New Phytol..

[22] Yang S.U., Kim H., Kim R.J., Kim J., Suh M.C. (2020). AP2/DREB Transcription Factor RAP2.4 Activates Cuticular Wax Biosynthesis in Arabidopsis Leaves Under Drought. Front. Plant Sci..

[23] Seo P.J., Lee S.B., Suh M.C., Park M.J., Go Y.S., Park C.M. (2011). The MYB96 transcription factor regulates cuticular wax biosynthesis under drought conditions in Arabidopsis. Plant Cell.

[24] Lee H.G., Kim J., Suh M.C., Seo P.J. (2017). The MIEL1 E3 Ubiquitin Ligase Negatively Regulates Cuticular Wax Biosynthesis in Arabidopsis Stems. Plant Cell Physiol..

[25] Lam P., Zhao L., Eveleigh N., Yu Y., Chen X., Kunst L. (2015). The exosome and trans-acting small interfering RNAs regulate cuticular wax biosynthesis during Arabidopsis inflorescence stem development. Plant Physiol..

[26] Lam P., Zhao L., McFarlane H.E., Aiga M., Lam V., Hooker T.S., Kunst L. (2012). RDR1 and SGS3, Components of RNA-Mediated Gene Silencing, Are Required for the Regulation of Cuticular Wax Biosynthesis in Developing Inflorescence Stems of Arabidopsis. Plant Physiol..

[27] Kim H., Yu S.-I., Jung S.H., Lee B.-H., Suh M.C. (2019). The F-Box Protein SAGL1 and ECERIFERUM3 Regulate Cuticular Wax Biosynthesis in Response to Changes in Humidity in Arabidopsis. Plant Cell.

[28] Ménard R., Verdier G., Ors M., Erhardt M., Beisson F., Shen W.-H. (2014). Histone H2B monoubiquitination is involved in the regulation of cutin and wax composition in *Arabidopsis thaliana*. Plant Cell Physiol..

[29] Lee S., Fu F., Xu S., Lee S.Y., Yun D.J., Mengiste T. (2016). Global Regulation of Plant Immunity by Histone Lysine Methyl Transferases. Plant Cell.

[30] Wang T., Xing J., Liu X., Yao Y., Hu Z., Peng H., Xin M., Zhou D.X., Zhang Y., Ni Z. (2018). GCN5 contributes to stem cuticular wax biosynthesis by histone acetylation of CER3 in Arabidopsis. J. Exp. Bot..

[31] Batool M., El-Badri A.M., Hassan M.U., Haiyun Y., Chunyun W., Zhenkun Y., Jie K., Wang B., Zhou G. (2023). Drought Stress in *Brassica napus*: Effects, Tolerance Mechanisms, and Management Strategies. J. Plant Growth Regul..

[32] Khanzada H., Wassan G.M., He H., Mason A.S., Keerio A.A., Khanzada S., Faheem M., Solangi A.M., Zhou Q., Fu D. (2020). Differentially evolved drought stress indices determine the genetic variation of *Brassica napus* at seedling traits by genome-wide association mapping. J. Adv. Res..

[33] Wang Y., Jin S., Xu Y., Li S., Zhang S., Yuan Z., Li J., Ni Y. (2020). Overexpression of *BnKCS1-1*, *BnKCS1-2*, and *BnCER1-2* promotes cuticular wax production and increases drought tolerance in *Brassica napus*. Crop J..

[34] Yamaguchi-Shinozaki K., Shinozaki K. (2006). Transcriptional regulatory networks in cellular responses and tolerance to dehydration and cold stresses. Annu. Rev. Plant Biol..

[35] Kosma D.K., Bourdenx B., Bernard A., Parsons E.P., Lü S., Joubès J., Jenks M.A. (2009). The impact of water deficiency on leaf cuticle lipids of Arabidopsis. Plant Physiol..

[36] Barthlott W., Mail M., Bhushan B., Koch K. (2017). Plant surfaces: Structures and functions for biomimetic innovations. Nano Micro Lett..

[37] Zhang X., Henriques R., Lin S.S., Niu Q.W., Chua N.H. (2006). *Agrobacterium*-mediated transformation of *Arabidopsis thaliana* using the floral dip method. Nat. Protoc..

[38] Jach G., Binot E., Frings S., Luxa K., Schell J. (2001). Use of red fluorescent protein from Discosoma sp. (dsRED) as a reporter for plant gene expression. Plant J..

[39] Sparkes I.A., Runions J., Kearns A., Hawes C. (2006). Rapid transient expression of fluorescent fusion proteins in tobacco plants and generation of stably transformed plants. Nat. Protoc..

[40] Zhou F., Lin Q., Zhu L., Ren Y., Zhou K., Shabek N., Wu F., Mao H., Dong W., Gan L. (2013). D14-SCF(D3)-dependent degradation of D53 regulates strigolactone signalling. Nature.

[41] Tang J., Yang X., Xiao C., Li J., Chen Y., Li R., Li S., Lü S., Hu H. (2020). GDSL lipase occluded stomatal pore 1 is required for wax biosynthesis and stomatal cuticular ledge formation. New Phytol..

[42] Kumar S., Stecher G., Tamura K. (2016). MEGA7: Molecular Evolutionary Genetics Analysis Version 7.0 for Bigger Datasets. Mol. Biol. Evol..

