# Supplementary material for: Brassica napus BnaC9.DEWAX1 Negatively Regulates Wax Biosynthesis via Transcriptional Suppression of BnCER1-2
Source: Int J Mol Sci. 2023 Feb 21;24(5):4287. doi: 10.3390/ijms24054287 (PMC10002155; doi:10.3390/ijms24054287)
Supplement: Supplementary file 1 [file ijms-24-04287-s001.zip › ijms-2222051-supplementary.pdf]

Figure S1 Interaction analysis of *BnCER1-2* promoter with BnaC9.DEWAX1 protein by yeast one-hybrid assay. (A) Nucleotide sequence of promoter region of *BnCER1-2* gene used in yeast one-hybrid assay, which is 100 bp upstream of the start codon of the *BnCER1-2* gene. Nucleotide in red indicated GCC-like motif. (B) Co-transfection verification for *BnCER1-2* promoter interaction with BnaC9.DEWAX1 protein. The prey and bait constructs were co-transformed to Y1H gold yeast competent cell. The prey construct contains the BnaC9.DEWAX1 gene driven by the CaMV 35S promoter. The bait construct consists of the AUR1-C antibiotic resistance gene driven by the *BnCER1-2* promoter linked with the yeast iso-1-cytochrome C minimal promoter.

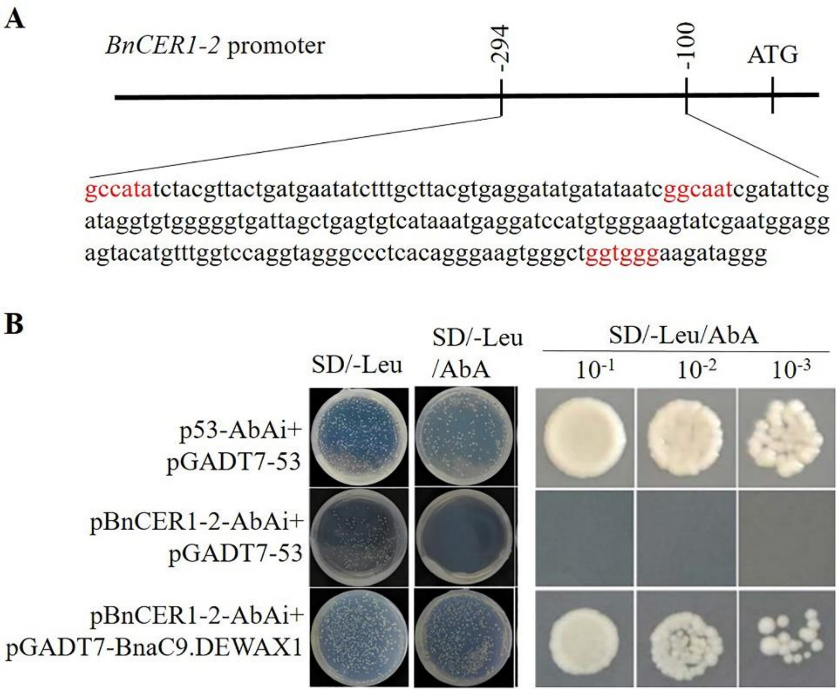

Figure S2 Nucleotide and amino acid sequences (A) and phylogenetic analysis (B) of BnaC9.DEWAX1. (A) The AP2/ERF DNA-binding domain is underlined, the putative nuclear localization signal (NLS) are double underlined, and the putative acidic domain is marked with dots below. (B) A phylogenetic tree constructed using BnaC9.DEWAX1 and other Arabidopsis ERF subfamily members (B-1~B-6). BnaC9.DEWAX1, indicated by the asterisk, was grouped with members of the ERF subfamily B-3, and sub-grouped with Arabidopsis DEWAX.

A

```
ATGACAACTTTTGAGAAAAGCTCTGATGTGGAGGTTATACACAAGTATCTCTTTGAGGAC  60
M  T  T  F  E  K  S  S  D  V  E  V  I  H  K  Y  L  F  E  D    20
TTGATGATCCCTGATTGCTTCATGGAAGATTTTGTCTTTGATGATGCTGCTTTTGTCTCA  120
L  M  I  P  D  C  F  M  E  D  F  V  F  D  D  A  A  F  V  S    40
GGACTCTTGTCTCTAGAACCCTTTAACCCAGTTCCTAAACAAGAGCCTAGTTCACCGGTT  180
G  L  L  S  L  E  P  F  N  P  V  P  K  Q  E  P  S  S  P  V    60
CTTGATCCAGATTCCTGTGTCCAAGAGTTTCTACAAATTGAAGCAGAATCATCATCATCG  240
L  D  P  D  S  C  V  Q  E  F  L  Q  I  E  A  E  S  S  S  S    80
TCAACAACTACAACATCACCTGAGGCTGAGACAGTCTCAAACCGAAAAAGACCAAGGAGG  300
S  T  T  T  T  S  P  E  A  E  T  V  S  N  R  K  R  P  R  R    100
GTCGAAGAGACGAGACACTACAGAGGCGTGAGAAGGAGACCATGGGGGAAATTTGCAGCA  360
V  E  E  T  R  H  Y  R  G  V  R  R  R  P  W  G  K  F  A  A    120
GAGATTCGAGATCCGGCCAAGAAAGGATCTAGGATGTGGCTAGGCACATTTGAGACTGAT  420
E  I  R  D  P  A  K  K  G  S  R  M  W  L  G  T  F  E  T  D    140
ATTGATGCTGCAAGAGCTTATGACTACACAGCTTTTAAGCTCAGGGGAAGAAAAGCTGTG  480
I  D  A  A  R  A  Y  D  Y  T  A  F  K  L  R  G  R  K  A  V    160
CTCAACTTTCCTTTGGATGCTGGTAAGTATGATGCTCCGATCAATTCTTGCCGGAAGAGG  540
L  N  F  P  L  D  A  G  K  Y  D  A  P  I  N  S  C  R  K  R    180
AGAAGAAACGATGTACCGGAGCCTCAAGGAATAACTACTAGTACTACATCATCTTCATCA  600
R  R  N  D  V  P  E  P  Q  G  I  T  T  S  T  T  S  S  S  S    200
AACTAA                                                        606
N  *                                                          201
```

B

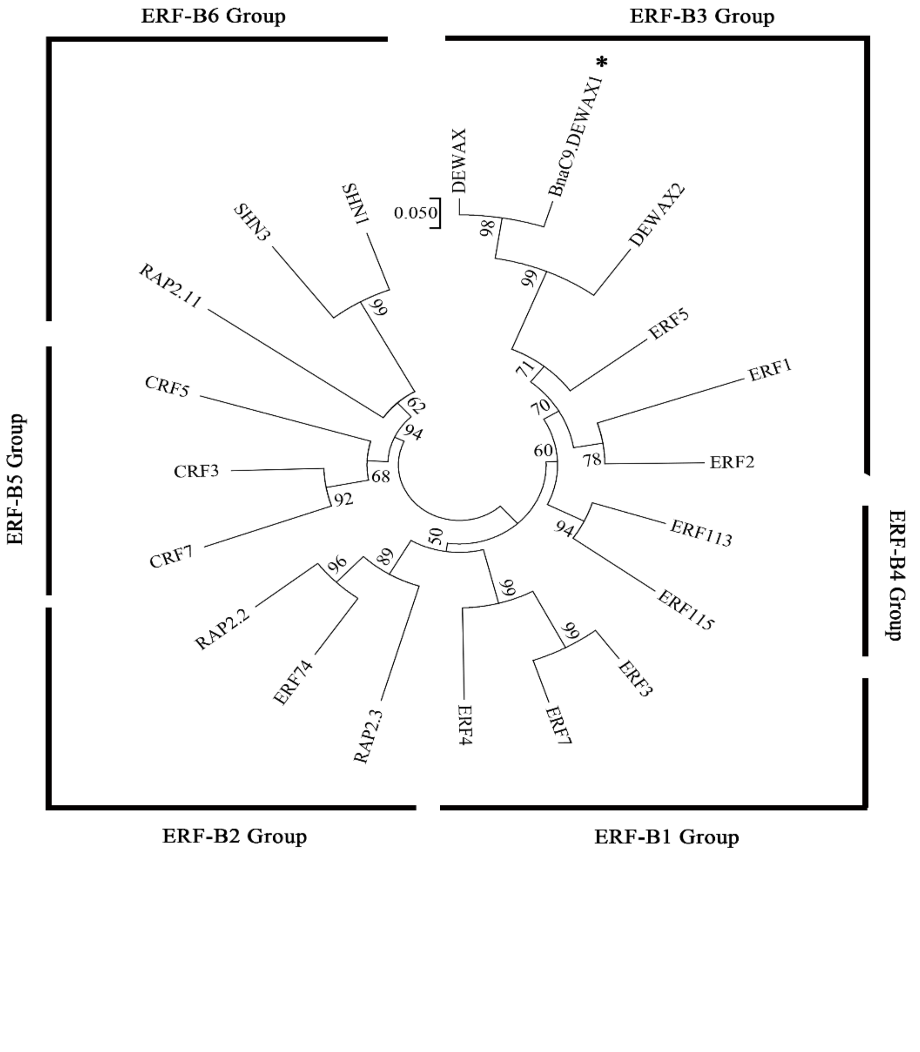

Table S1 Nucleotide sequence in *BnCER1-2* and *CER1* promoter regions regulated by BnaC9.DEWAX1.

Genes	Position (relative to ATG)	Nucleotide sequences of promoter regions (5'-3')
BnCER1-2	-294 ~ -100	gccatatctacgttactgatgaatatctttgcttacgtgaggatatgatataatcggcaatcgatattcgataggtgtgggggtgattagctgagtgtcataaatgaggatccatgtgggaagtatcgaatggaggagtacatgtttggtccaggtagggccctcacagggaagtgggct**ggtggg**aagataggg
CER1	-317 ~ -153	gaagccatccatcaaccggtgcatttcctcaaggcatggatatgatatcagaacatcgatgaa**ggtgg** **g**agggggtaattagctgagtgtcataaatgaggatccatgtggagatcatcgaatggtagtagtacatgtttggtcttagctggccccaccacaag

Red indicated GCC-like motif.

Table S2 Primers used in this study.

**Name**	**Sequence (5'-3')**	
**For qRT-PCR**		
BnDEWAX1-Fq	GTTCTTGATCCAGATTCCTGT	
BnDEWAX1-Rq	GTGATGTTGTAGTTGTTGACG	
BnACT7-Fq	GTGACAATGGAACTGGAATGGTGA	
BnACT7-Rq	GTGCCTAGGACGACCAACAATACTC	
CER1-Fq	AGGTCGACAGGGAGACCAAC	
CER1-Rq	ATAAGCGCTGCCATCAACAC	
AtEF1a-qF	TCGTTATGATCGACTCTCTTATGG	
AtEF1-qR	CCAAAAAGGAGGGAGAGAGAAAG	
**For subcellular localization**		
BnDEWAX1-Fs	GGAATTCATGACAACTTTTGAGAAAAGCTCTG	*Eco*RI
BnDEWAX1-Rs	CGGGATCCTTAGTTTGATGAAGATGATGTAGTA	*Bam*HI
**For overexpression/complementation transgenic arabidopsis**		
BnDEWAX1-Fo	CATTTGGAGAGGACACTAGTGGATCCATGACAACTTTTGAGAAAAGCTCTG	*Bam*HI
BnDEWAX1-Ro	GCAGGGTCGACGGTATCGATAAGCTTTTAGTTTGATGAAGATGATGTAGTA	*Hin*dIII
Hpt-F	CTGAACTCACCGCGACGTCTGTC	
Hpt-R	GCCTCCGCTCGAAGTAGCGC	
**Yeast one-hybrid assays**		
BnDEWAX1-Fy	CGGGATCCATGACAACTTTTGAGAAAAGCTCTG	*Bam*HI
BnDEWAX1-Ry	CCGCTCGAGTTAGTTTGATGAAGATGATGTAGTACTAGTAG	*Xho*I
BnCER1-2P-Fy	CCCAAGCTTGCCATATCTACGTTACTGATGAATATC	*Hin*dIII
BnCER1-2P-Ry	CCGCTCGAGCCCTATCTTCCCACCAGCC	*Xho*I
**For transcriptional activity assay**		
BnDEWAX1-Fra	CCGTCTAGAACTAGTGGATCCATGACAACTTTTGAGAAAAGCTCTG	*Bam*HI
BnDEWAX1-Rra	TTTGCGGAGTACCCGGGTACCTTAGTTTGATGAAGATGATGTAGTA	*Kpn*I
**For Transient dual-LUC assay**		
BnDEWAX1-Ftr	GGAATTCATGACAACTTTTGAGAAAAGCTCTG	*Eco*RI
BnDEWAX1-Rtr	CCGCTCGAGTTAGTTTGATGAAGATGATGTAGTACTAGTAG	*Xho*I
BnCER1-2P-Ftr	GTCGACGGTATCGATAAGCTTGCCATATCTACGTTACTGATGAATATC	*Hin*dIII
BnCER1-2P-Rtr	AGAACTAGTGGATCCCCCGGGCCCTATCTTCCCACCAGCC	*Sma*I
**For porokaryotic expression**		
BnDEWAX1-Fgex	CCGCGTGGATCCCCGGAATTCATGACAACTTTTGAGAAAAGCTCTG	*Eco*RI
BnDEWAX1-Rgex	GTCACGATGCGGCCGCTCGAGCTACTGAGGAGGATTCATCTCCGGT	*Xho*I

The underlined indicates the site of restrication enzyme.
